# IFN-β Plays Both Pro- and Anti-inflammatory Roles in the Rat Cardiac Fibroblast Through Differential STAT Protein Activation

**DOI:** 10.3389/fphar.2018.01368

**Published:** 2018-11-28

**Authors:** Samir Bolívar, Renatto Anfossi, Claudio Humeres, Raúl Vivar, Pía Boza, Claudia Muñoz, Viviana Pardo-Jimenez, Francisco Olivares-Silva, Guillermo Díaz-Araya

**Affiliations:** ^1^Faculty of Chemistry and Pharmacy, Atlantic University, Barranquilla, Colombia; ^2^Department of Chemical Pharmacology and Toxicology, Faculty of Chemical and Pharmaceutical Sciences, University of Chile, Santiago, Chile; ^3^Advanced Center for Chronic Diseases, Faculty of Chemical and Pharmaceutical Sciences and Faculty of Medicine, University of Chile, Santiago, Chile

**Keywords:** IFN-β (interferon β), cardiac fibroblast, STAT, proinflammatory, anti-infammatory

## Abstract

Cardiac fibroblasts (CFs) contribute to theinflammatory response to tissue damage, secreting both pro- and anti-inflammatory cytokines and chemokines. Interferon beta (IFN-β) induces the phosphorylation of signal transducer and activator of transcription (STAT) proteins through the activation of its own receptor, modulating the secretion of cytokines and chemokines which regulate inflammation. However, the role of IFN-β and STAT proteins in modulating the inflammatory response of CF remains unknown. CF were isolated from adult male rats and subsequently stimulated with IFN-β to evaluate the participation of STAT proteins in secreting chemokines, cytokines, cell adhesion proteins expression and in their capacity to recruit neutrophils. In addition, in CF in which the TRL4 receptor was pre-activated, the effect of INF-β on the aforementioned responses was also evaluated. Cardiac fibroblasts stimulation with IFN-β showed an increase in STAT1, STAT2, and STAT3 phosphorylation. IFN-β stimulation through STAT1 activation increased proinflammatory chemokines MCP-1 and IP-10 secretion, whereas IFN-β induced activation of STAT3 increased cytokine secretion of anti-inflammatory IL-10. Moreover, in TLR4-activated CF, IFN-β through STAT2 and/or STAT3, produced an anti-inflammatory effect, reducing pro-IL-1β, TNF-α, IL-6, MCP-1, and IP-10 secretion; and decreasing neutrophil recruitment by decreasing ICAM-1 and VCAM-1 expression. Altogether, our results indicate that IFN-β exerts both pro-inflammatory and anti-inflammatory effects in non-stimulated CF, through differential activation of STAT proteins. When CF were previously treated with an inflammatory agent such as TLR-4 activation, IFN-β effects were predominantly anti-inflammatory.

## Introduction

The classical role of cardiac fibroblasts (CFs) is extracellular matrix (ECM) protein homeostasis. However, CF can also act as “sentinel” cells, detecting changes in chemical and mechanical signals in the heart and stimulating an appropriate response (Frangogiannis, 2007; Van Linthout et al., 2014; Díaz-Araya et al., 2015). Immediately after tissue damage, CF contribute to the inflammation necessary for repair, secreting pro-inflammatory cytokines, such as tumor necrosis factor alpha (TNF-α) and interleukin 1 beta (IL-1β), as well as pro-inflammatory chemokines, such as monocyte chemoattractant protein 1 (MCP-1) and interleukin-8 (Kukielka et al., 1995). However, CF also secrete inhibitory mediators capable of suppressing pro-inflammatory signals, such as interleukin-10 (IL-10) and transforming growth factor beta (TGF-β) (Dewald et al., 2005; Nahrendorf et al., 2007; Turner et al., 2009, 2010). Finally, CF express intercellular and vascular adhesion molecules (ICAM-1, VCAM-1, and E-selectin) to recruit immune cells (Kacimi et al., 1998; Turner et al., 2011).

Interferon beta (IFN-β) is a cytokine produced by innate immune cells, including macrophages and dendritic cells, as well as non-immune cells, such as fibroblasts and epithelial cells (Ivashkiv and Donlin, 2014). IFN-β elicits a wide range of effects, including anti-inflammatory and pro-inflammatory responses, and also regulates the development and activation of virtually all innate and adaptive immune effector cells. IFN-β has a variety of documented effects on immune cells; for instance, IFN-β modulates TNF-α and IL-10 expression in peripheral blood mononuclear cells (Rudick et al., 1996; Coclet-Ninin et al., 1997), reduces TNF-α expression in monocytes (Jungo et al., 2001), and increases IL-10 expression in dendritic cells (Yen et al., 2015). However, the effects of IFN-β on non-immune cells such as CF remain unknown.

The cellular effects of IFN-β are mediated through the JAK/STAT signaling pathway and vary according to context and cell phenotype (Darnell et al., 1994; Ihle, 1995; Chen et al., 2004). Interferon type I receptor (IFNAR)activation by IFN-β induces signal transducer and activator of transcription (STAT) protein phosphorylation, and the canonical IFN-β signaling pathway can activate the formation of p-STAT1/p-STAT2 heterodimers that bind to interferon regulator factor 9. The activated proteins then translocate to the nucleus, activating the transcription of inflammatory genes known as interferon-stimulated response elements (Platanias, 2005). In contrast, the formation of p-STAT1 and/or p-STAT3 homodimers inhibits the activation of p-STAT1/p-STAT2 heterodimer-dependent genes, suppressing the inflammatory properties of IFN-β (Ivashkiv and Donlin, 2014; López de Padilla and Niewold, 2016). Whereas, recent studies have provided significant insight into the roles of activated STAT2 and STAT3 proteins in modulating inflammation, especially in immune and vascular cells (Szelag et al., 2016). STAT3 modulates proliferation, differentiation, survival, oxidative stress, and metabolism in cardiomyocytes, fibroblasts, endothelial cells, progenitor cells, and various inflammatory cells (O’Shea and Murray, 2008; Haghikia et al., 2014). However, the effects of STAT protein activation by IFN-β on modulation of inflammatory response in CF are unclear. In this work, we sought to study the effects of IFN-β on CF pro-inflammatory (MCP-1, IL-1β, TNF-α and IP-10 secretion; VCAM-1 and ICAM-1 expression) and anti-inflammatory effects (IL-10 secretion); and the involvement of STAT pathway in mediating these actions. Furthermore, we hypothesized that these effects could be greatly influenced by inflammatory activation of CF, for which we stimulated CF TLR4 receptor, a well-studied receptor involved in pro-inflammatory fibroblast functions, with lipopolysaccharide (LPS), a TLR4 specific agonist, to promote CF inflammatory environment.

## Materials and Methods

### Materials

Fetal bovine serum (FBS), trypsin/EDTA and prestained molecular weight marker were purchased from Merck (Darmstadt, Germany). Enhanced chemiluminescence (ECL) reagent was acquired from PerkinElmer Life Sciences (Boston, MA, United States). Sterile plastic material was purchased from CoStar (Corning Costar, NJ, United States). Mouse primary antibodies for ICAM-1, and VCAM-1 were purchased from Abcam (Cambridge, MA, United States). Rabbit GAPDH was acquired from Calbiochem (Darmstadt, Germany). Anti-RP1 PE Mouse anti-rat granulocytes was purchased from BD Biosciences (Franklin Lakes, NJ, United States). Fluorescent-conjugated goat anti-mouse IgG (H+L) secondary antibodies, Alexa Fluor 488 and 555 and Opti-MEM^®^ were purchased from Thermo Fisher Scientific (Waltham, MA, United States). HRP-conjugated antibodies were purchased from Santa Cruz Biotechnology (Santa Cruz, CA, United States). The primary antibodies for p-Stat1, p-Stat2, and p-Stat3 were purchased from Cell Signaling Technology (Boston, MA, United States). Anti- Stat1, Stat2 and Stat3 were obtained from Santa Cruz Biotechnology (Dallas, TX, United States). Ficoll Histopaque-1083 was purchased from Sigma-Aldrich (St. Louis, MO, United States). Multiplex kit (RECYTMAG 65K/MILLIPLEX MAP Rat Cytokine/Chemokine Magnetic Bead Panel) and TGF-β1 were purchased from Merck-Millipore (Tamecula, CA, United States). Lipofectamine^®^ 2000 was purchased from Invitrogen (Carlsbad, CA, United States). Transwellfilename plates (#3422) were purchased from Corning (New York, NY, United States). The Silencer^®^ against Stat1, Stat2, Stat3 and Scramble siRNA (negative control Silencer^®^) were obtained from Life Technologies. InvivoGen LPS-EB Ultrapure (InvivoGen tlrl-pelps, from *E. coli* O111:B4) was extracted through successive hydrolysis steps and purified by phenol-TEA-DOC extraction; according to InvivoGen, this LPS is specific for TLR4 activation. Collagenase type II was purchased from InvivoGen (San Diego, CA, United States).

### Animals

Male adult Sprague Dawley rats were obtained from the animal breeding facility of the Faculty of Chemical and Pharmaceutical Sciences at the University of Chile. The animals were housed in cages (12-h light/dark cycles) with access to rat chow and water ad libitum. All studies were performed in compliance with the NIH Guide for the Care and Use of Laboratory Animals, updated in 2011^1^, and experimental protocols were approved by our Institutional Ethics Review Committee.

### Cell Culture and Treatments

Cardiac fibroblast were isolated from adult Sprague Dawley Rats (3–4 weeks old) using enzymatic digestion, as previously described (Humeres et al., 2016). Briefly, rats were anesthetized by ketamine/xylazine injection and their hearts extracted in an aseptic environment. Atria were removed and ventricles cut into small pieces (1–2 mm) for later collagenase II digestion. The digestion yield was separated by 10-min centrifugation at 1000 rpm. The pellet was resuspended in 10 ml of Dulbecco’s Modified Eagle Medium/Nutrient Mixture F-12 (DMEM/F12) supplemented with 10% FBS and antibiotics (100 μg/ml streptomycin and 100 units/ml penicillin) and cultured in a humid atmosphere at 5% CO_2_/95% O_2_ and 37°C until confluence (5 days). The purity of the CF population was assessed with several markers. CF showed positive staining for vimentin (Santa Cruz Biotechnology, Santa Cruz, CA, United States) and were negative against sarcomeric actin and desmin (Sigma-Aldrich, St. Louis, MO, United States).

Cells from passage 1 were used for all experiments, and they were maintained in DMEM F12 for 18 h until experiments were performed. CF were pre-treated with IFN-β (500 U/mL) for 30 to 120 min, and LPS (1 μg/mL) was used as inflammatory stimulus. For experiments involved with JAK/STAT inhibition, CF were incubated with Ruxolitinib (JAK inhibitor, 500 nM), or transfected with Stat1, Stat2, and Stat3 siRNAs (100 ng/mL) for 8 h previous Ruxolitinib, IFN-β or LPS incubation.

### Isolation of Neutrophils From Bone Marrow

For neutrophils isolation from bone marrow, we used cell sorting by flow cytometry fluorescence strategy (FACS) (BD FACSAria^TM^ III, BD Biosciences) using RP-1 antibody as specific marker for rat neutrophils (Skrajnar et al., 2009). The femur and tibia were extracted from both extremities of the rats, and the epiphysis was excised at the level of the metaphysis of both bone types. The bone marrow is then removed by infusion of DMEM/F12 medium by means of a 25G-wide syringe onto a 100 mm Petri dish. The leukocyte and erythrocyte rich cell suspension was then centrifuged at 300 *g* for 10 min at room temperature. The erythrocytes present in the suspension were then lysed with 0.83% ammonium chloride (NH_4_Cl) for 7 min and the remaining suspension was washed twice with tissue storage solution (MACS) (2 mM EDTA, 0.5% BSA) and incubated with phycoerythrin (PE) conjugated anti RP-1 antibody at a concentration of 0.5 μl anti-RP-1 per 1 × 10^6^ cells in 100 μl of total volume for 30 min, avoiding light. The leukocyte suspension is then washed twice with MACS medium and assayed by Cell Sorting (Beckson-Dickinson FACS flow and sorting cytometer). Approximately 20% of cells isolated were identified as RP-1 + cells (neutrophils). The sorted population consisted of 98% RP-1 positive neutrophils (less than 1–2% monocytes/lymphocytes) and a 99% in viability. Purified neutrophils were centrifuged and pooled for further experiments.

### STATs Knockdown

FC were plated in 35 mm to 80% confluency and serum starved overnight. A solution of 4 uL Lipofectamine^®^ + 100 uL Opti-MEM^®^ and 5 uL siRNA (Silencer^®^) + 100 uL Opti-MEM^®^ was incubated for 15 min at room temperature, mixed and further incubated for 15 min. CF were treated with the siRNA preparation for 8 h at 37°C, media replaced DMEM F12 for additional 24 h and lastly cells were harvested for further analysis. The codes products used were: RefSeq Stat1 NM_001034164.1, RefSeq Stat2 NM_001011905.1, RefSeq Stat3 NM_012747.2.

### Western Blot Analysis

After respective treatments, cells were lysed in 50 mM Tris, 300 mM NaCl, 1 mM MgCl_2_, 0.5 mM ethylenediaminetetraacetic acid (EDTA), 0.1 mM EGTA, 20% glycerol, 1% NP40 cell lysis buffer, 0.5 mM 1,4-dithiothreitol (DTT), and inhibitor cocktail. Lysates were vigorously vortexed for 10 s and centrifuged at 15,000 rpm for 10 min, and total protein content was determined using Bradford assay. Equivalent amounts of protein were loaded to SDS PAGE. Western blotting was performed by transferring proteins to nitrocellulose membranes and blocking with 10% fat-free milk (w/v) in TBS-Tween for 1 h at room temperature. Membranes were probed with the appropriate primary antibody overnight at 4°C and then with peroxidase-conjugated secondary antibody for 2 h at room temperature. Finally, the ECL Advance Western Blotting Detection Kit was used for immunodetection. Protein levels were determined by densitometric analysis using Image J (NIH, Bethesda, MD, United States) and normalized to GAPDH and total STAT levels, according to the experiment.

### Immunofluorescence Assay

Cardiac fibroblasts were fixed in 4% paraformaldehyde solution for 20 min at room temperature and permeabilized in 0.1% Triton X-100 for 10 min at room temperature. Non-specific proteins were blocked with 3% bovine serum albumin solution for 30 min at room temperature. Cells in coverslips were incubated with the appropriate primary antibody overnight at 4°C and an appropriate fluorophore-conjugated secondary antibody for 2 h at room temperature. Images were obtained using a spinning-disk microscope.

### Luminex Immunobead Assay

Supernatant was collected and stored at -80°C prior to cytokine analysis. Depending on the experiment CF were previously transfected with Stat siRNA and treated with IFN-β or LPS, or both for 24 h. One h before analysis, samples were thawed and analyzed for cytokine and chemokine protein levels using the Milliplex MAP-Rat Cytokine/Chemokine Magnetic Bead Panel Immunology Multiplex Assay according to manufacturer instructions. The proinflammatory cytokines (Il-6, TNF-α) and chemokines (MCP-1 and IP-10); and also antinflammatory cytoquines (IL-10) were measured. Cytokine levels were read on Luminex 200 System, Multiplex Bio-Assay. Quantification was based on standard curves for each cytokine, in the concentration range of 1.5–10,000 pg/ml. Values were standardized by measuring total amounts of cells and expressed as pg/50.000 cells.

### Migration Assays of Neutrophils

*In vitro* neutrophil migration assays were performed using Transwell^®^ plates with porous polycarbonate membrane filters (6.5 mm diameter, 8.0 μm pore) between an upper and lower compartment. In the upper chamber, 4 × 10^5^ neutrophils labeled with calcein were seeded in 200 μL of DMEM/F12. The lower chamber contained 500 μl of conditioned medium obtained from the treated CF with IFN-β and/or LPS in the presence or absence of anti-STAT siRNA. The neutrophils migrated for 3 h at 37°C and 5% CO_2_/95% O_2_. The fluorescently labeled neutrophils that migrated to the lower compartment were measured using a fluorescence spectrometer equipped with a microplate (Multi-Mode Reader Synergy 2, BioTek). DMEM/F12 (without CF supernatant) was used in the lower compartment as a negative control and DMEM/F12 + 50% FBS was used as a positive control. The percentage of neutrophil migration was calculated as follows; 100 x [fluorescence of migrated cells/fluorescence of total aggregated cells]. Prior to these studies, we performed neutrophil population identification tests. RP-1 protein expression results confirmed that 20% of the total leukocyte population was composed of neutrophils (Supplementary Figure S1).

### Adhesion Assays of Neutrophils

Cell adhesion *in vitro* was analyzed by Cell Sorting (Beckson-Dickinson FACS flow and sorting cytometer). In brief, leukocytes obtained from rat bone marrow were resuspended in DMEM/F12 medium at a rate of 1.5 × 10^6^ cells/100 μl, and then were added to 35 mm plates on a confluent monolayer of CF (15 × 10^4^) previously transfected with and without Scramble, siRNA Stat1, siRNA Stat2, and siRNA Stat3, and treated in the presence or absence of LPS and IFN-β. After an incubation period of 2 h at 37°C, cell plates were washed with 1X sterile phosphate-buffered saline (PBS) and CF with adhered leukocytes were peeled off using 0.1% trypsin in 1X sterile PBS. The suspension of CF and adhered leukocytes were then washed with MACS medium for 5 min at room temperature and incubated with anti-RP-1/PE antibody at the rate of 0.5 μl of anti-RP-1 per 10^6^ cells in 100 μl of total volume for 30 min, avoiding light. The cell suspension (CF and leukocyte), was then washed twice with MACS medium and assayed by Cell Sorting. Percentage of neutrophil adhesion was calculated as follows; 100 x (number of adherent RP-1 + cells/total number of aggregated RP-1 + cells). The analysis was performed using FlowJo 7.0 (TreeStar Software).

### Statistical Analysis

Data are expressed as the mean ± SD of at least three independent experiments. Statistical analysis was performed using one-way ANOVA followed by Tukey’s multiple comparison tests with GraphPad Prism Software. *p* < 0.05 was considered significant.

## Results

### IFN-β Activates the JAK/STAT Signaling Pathway in CF

**FIGURE 1:**
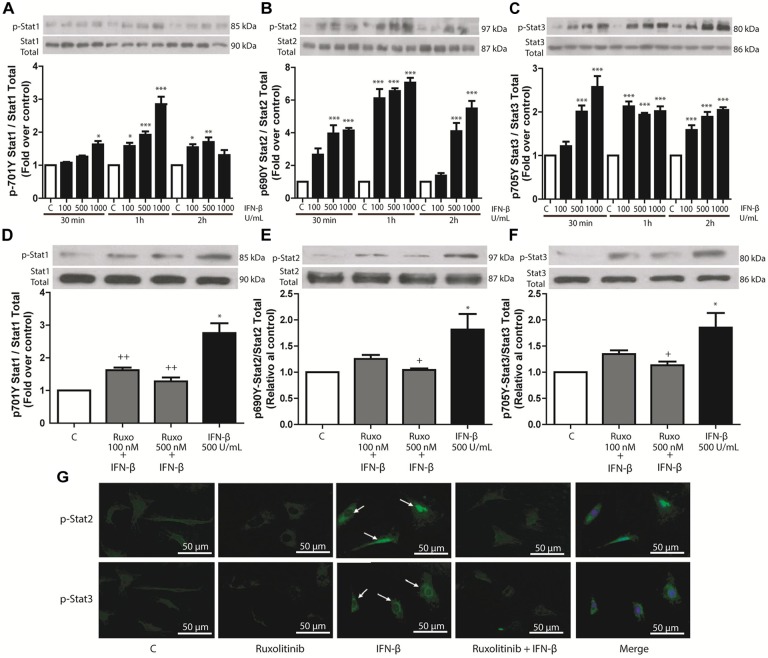
IFN-β activates the JAK/STAT signaling pathway in CF. **(A–C)** The upper panelshows a Western blot image, indicating the INF-β-induced p-STAT1, p-STAT2, and p-STAT3 expression levels; STAT1, STAT2, and STAT3 were used as loading controls. The graphical analysis is presented at the bottom of each panel. Error bars indicate theSD for four independent experiments. ^∗∗∗^*p* < 0.001, ^∗∗^*p* < 0.01, ^∗^*p* < 0.05 vs. control. **(D–F)** CF were pretreated with or without Ruxolitinib (100 and 500 nM) for 1 h and stimulated with IFN-β (500 U/ml) for 24 h. The upper panel of each figure corresponds to the representative image of p-STAT1, p-STAT2, and p-STAT3 expression levels; STAT1, STAT2, and STAT3 were used as loading controls. The graphical analysis is presented at the bottom of each panel. Error bars indicate the SD for four independent experiments. ^∗∗∗^*p* < 0.001, ^∗^*p* < 0.05 vs. control. ^++^*p* < 0.01, *p* < 0.05 vs. IFN-β. **(G)** CF were pretreated with or without Ruxolitinib (100 and 500 nM) for 1 h and stimulated with IFN-β (500 U/ml) for 24 h. p-STAT2 and p-STAT3 were detected by immunofluorescence using anti-p-STAT2 and anti-p-STAT3 antibodies and Alexa Fluor^®^ 488-conjugated secondary antibody (green staining). Representative images of the immunocytochemistry for three independent experiments are shown, indicating the nuclear translocation of p-STAT2 and p-STAT3. ^+^*p* < 0.05 vs. IFN-β.

IFN-β (100, 500, or 1000 U/mL) induced concentration-dependent phosphorylation of STAT1, STAT2, and STAT3 in CF. Results were significant after 30 min and 1 and 2 h of IFN-β stimulation, respectively (Figures 1A–C). To assess whether STAT phosphorylation was dependent on the activation of JAK/STAT signaling pathway by IFN-β through activation of IFN-β receptor (IFNAR), CF were pretreated with 100 or 500 nM of JAK inhibitor Ruxolitinib, which inhibited IFN-β-induced STAT1, STAT2, and STAT3 protein phosphorylation (Figures 1D–F). Immunofluorescence results were consistent with this finding (Figure 1G); which showed that stimulation of CF with IFN-β (500 U/mL) for 1 h increased STAT2 and STAT3 nuclear localization (white arrows). Taken together, these results show that IFN-β activates the JAK/STAT signaling pathway through the IFNAR.

### In TLR4-Activated CF, IFN-β Inhibits Cytokines and Chemokines Secretion

**FIGURE 2:**
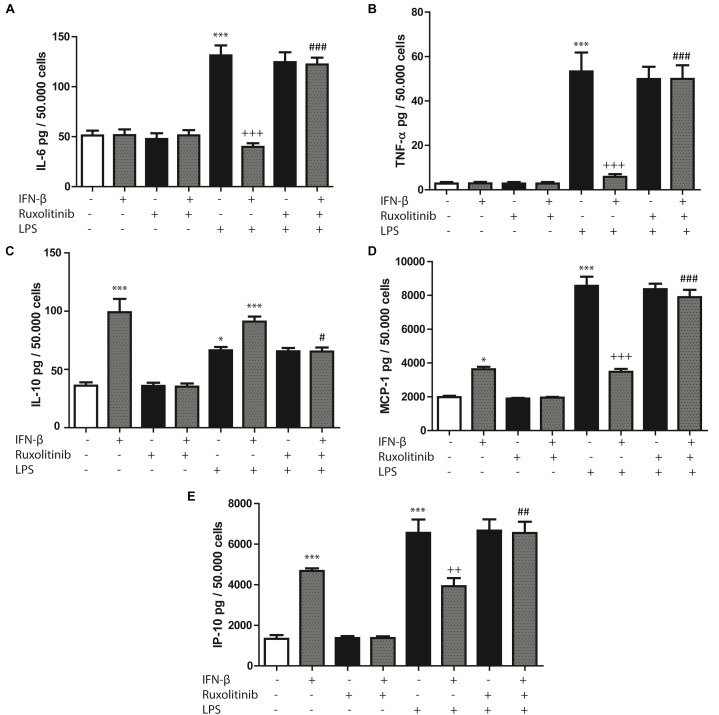
Effects of IFN-β and STAT proteins on LPS-induced cytokine secretion: **(A–E)** CF were transfected with scramble or 200 ng of si-STAT1, si-STAT2, and si-STAT3 for 8 h, serum-deprived of for 24 h, pre-treated with IFN-β for 1 h, and stimulated with LPS for 24 h. **(A)** IL-6, **(B)** TNF-β, **(C)** IL-10, **(D)** MCP-1 and **(E)** IP-10 secretion to the culture medium was determined by LUMINEX assay. Error bars indicate the SD for three independent experiments. ^∗∗∗^*p* < 0.001, ^∗∗^*p* < 0.01, vs. scramble. ^+++^*p* < 0.001 vs. scramble + LPS. ^###^*p* < 0.001, ^##^*p* < 0.01 vs. scramble + IFN-β + LPS. ^&&&^*p* < 0.001, ^&&^*p* < 0.01 vs. scramble + IFN-β.

Cardiac fibroblasts are important cellular effectors of the inflammatory response in cardiac tissue, where TLR4 activation by LPS or heparan sulfate has been shown to induce cytokine and chemokine secretion. Therefore, we evaluated whether IFN-β stimulates cytokines and chemokines secretion by CF. CF treated with IFN-β (24 h) did not modify IL-6 or TNF-α secretion levels, whereas it increased IL-10 secretion (Figures 2A–C). Ruxolitinib significantly inhibited the increase of IL-10 secretion induced by IFN-β. In contrast, treating CF with LPS (24 h) significantly increased IL-6, TNF-α and IL-10 secretion, although LPS produced a smaller effect than IFN-β on IL-10 secretion. Pre-treating CF with IFN-β for 1 h and then LPS for 24 h significantly inhibited the LPS-induced increase in IL-6 and TNF-α secretion (Figures 2A,B). However, IL-10 secretion levels were similar to those for IFN-β, and Ruxolitinib inhibited the effects of IFN-β, in the presence of LPS (Figure 2C).

In regard to chemokines, treatment with either IFN-β or LPS increased MCP-1 and IP-10 secretion, although LPS produced a larger effect than IFN-β. However, pre-treating CF with IFN-β for 1 h and then LPS for 24 h resulted in lower MCP-1 and IP-10 secretion levels than those associated with LPS treatment. Pre-treatment with Ruxolitinib inhibited the effects of IFN-β in the presence or the absence of LPS (Figures 2D,E).

Collectively, these results suggest that in CF, IFN-β by itself stimulates IL-10, MCP-1 and IP-10 secretion; however, in the presence of a pro-inflammatory stimulus, such as LPS, INF-β dampens the secretion of inflammatory cytokines and chemokines triggered by this pro-inflammatory stimulus.

### IFN-β Activates STAT2 and/or STAT3 to Inhibit LPS-Induced Cytokine and Chemokine Secretion in CF

STAT1, STAT2, and STAT3 siRNA (si-STAT1, si-STAT2, si-STAT3) were used to evaluate whether IFN-β-activated STAT proteins modulated cytokine or chemokine secretion, either alone or in the presence of LPS. Each siRNA was specific for its particular target (Supplementary Figure S2).

**FIGURE 3:**
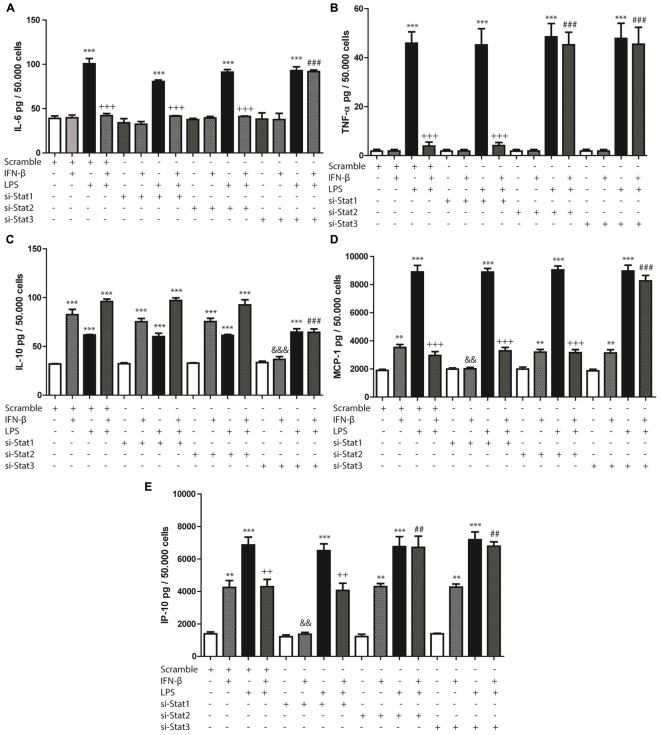
Effects of IFN-β and STAT proteins on LPS-induced cytokine secretion: **(A–E)** CF were transfected with scramble or 200 ng of si-STAT1, si-STAT2, and si-STAT3 for 8 h, serum-deprived of for 24 h, pre-treated with IFN-β for 1 h, and stimulated with LPS for 24 h. **(A)** IL-6, **(B)** TNF-β, **(C)** IL-10, **(D)** MCP-1 and **(E)** IP-10 secretion to the culture medium was determined by LUMINEX assay. Error bars indicate the SD for three independent experiments. ^∗∗∗^*p* < 0.001, ^∗∗^*p* < 0.01, vs. scramble. ^+++^*p* < 0.001 vs. scramble + LPS. ^###^*p* < 0.001, ^##^*p* < 0.01 vs. scramble + IFN-β + LPS. ^&&&^*p* < 0.001, ^&&^p*p* < 0.01 vs. scramble + IFN-β.

STAT3 activation modulated IFN-β-induced decrease of IL-6 in presence of LPS, since si-STAT3 prevented the decrease of IL-6 induced by IFN-β on LPS-treated CF. Both STAT2 and STAT3 activation were involved in IFN-β-induced decrease of TNF-α levels in LPS treated CF, as demonstrated by si-STAT2 and si-STAT3 prevention of TNF-α decrease. On the contrary, cytokine levels (IL-6 and TNF-α) induced by IFN-β on LPS-treated CF were unaffected by STAT1 knockdown (Figures 3A,B).

In addition, STAT3 (and not STAT1 or STAT2) was also crucial in IFN-β-induced IL-10 secretion, since STAT3 knockdown reverse IFN-β effects both in presence and absence of LPS (Figure 3C). In the case of MCP-1 and IP-10 secretion increased by IFN-β, it was observed that only STAT1 was necessary for such effect (Figures 3D,E). In contrast, STAT3 played an important role in modulating the IFN-β-induced MCP-1 secretion in the presence of LPS, since si-STAT3 reversed the effects of IFN-β on the LPS-induced activity. Neither STAT1 nor STAT2 modulated these effects of IFN-β (Figure 3D). Finally, in regard to IP-10 secretion, the effect of IFN-β in presence of LPS was dependent on both STAT2 and STAT3 activation, while being independent of STAT1 (Figure 3E).

### IFN-β Activates STAT2 and STAT3 to Modulate LPS-Induced Pro-IL-1β Expression in CF

**FIGURE 4:**
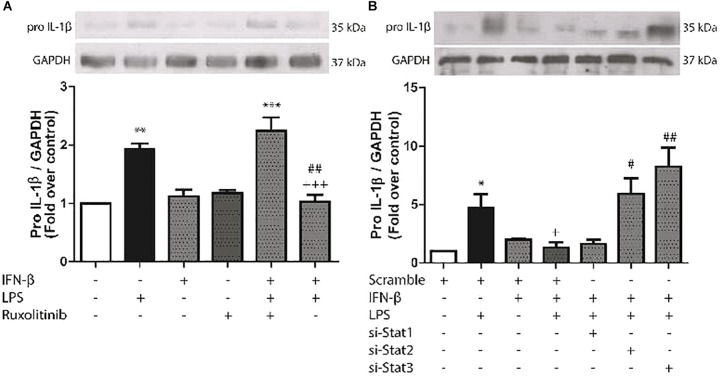
Effects of IFN-β and STAT proteinson LPS-induced pro-IL-1β protein expression. **(A)** CF were pretreated with or without Ruxolitinib (500 nM) for 1 h and stimulated with IFN-β (500 U/ml) for 24 h. The upper panel shows a representative Western blot image, indicating expression levels of pro-IL-1β protein and GAPDH (used as charge control). The graphical analysis is presented at the bottom of the panel. Error bars indicate the SD for three independent experiments. ^∗∗∗^*p* < 0.001, ^∗∗^*p* < 0.01 vs. control. ^##^*p* < 0.01 vs. LPS. ^+++^*p* < 0.001 vs. Ruxo + IFN-β + LPS). **(B)** CF were transfected with scramble or 200 ng of si-STAT1, si-STAT2, and si-STAT3 for 8 h, serum-deprived for 24 h, pretreated with IFN-β (500 U/ml) for 1 h, and stimulated with LPS (1 μg/ml) for 8 h. The upper panel shows a Western blot image, indicating expression levels of pro-IL-1β protein and GAPDH (used as charge control). The graphical analysis is presented at the bottom of the panel. Error bars indicate the SD for three independent experiments. ^∗^*p* < 0.05 vs. scramble. ^+^*p* < 0.05 vs. scramble + LPS. ^##^*p* < 0.01, ^#^*p* < 0.05 vs. scramble + IFN-β + LPS.

We have previously reported that LPS induces pro-IL-1β expression in CF. To evaluate the effects of IFN-β on expression of this cytokine, CF were treated with LPS for 8 h. LPS significantly increased pro-IL-1β expression, while IFN-β by itself had no effect. However, INF-β significantly decreased LPS-induced pro-IL-1β expression, which was completely abolished when CF were pretreated with Ruxolitinib (Figure 4A) As shown by the use of siRNA, both si-STAT2 and si-STAT3 were involved in IFN-β effects on pro-IL-1β expression on LPS-treated CF, whereas STAT1 was not involved (Figure 4B).

### IFN-β Activates STAT3 to Inhibit LPS-Induced ICAM-1 and VCAM-1 Expression in CF

**FIGURE 5:**
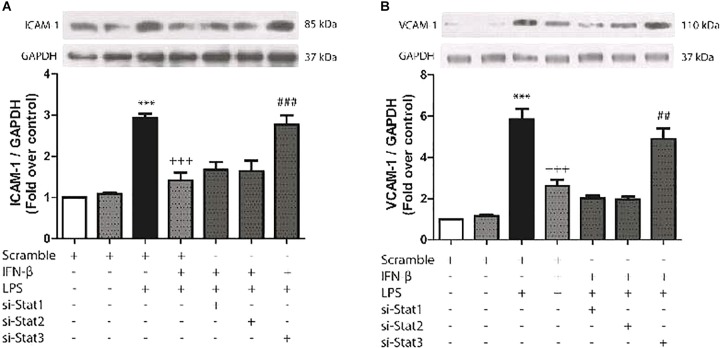
IFN-β decreases LPS-induced ICAM-1/VCAM-1 expression through STAT3. **(A–B)** CF were transfected with scramble or 200 ng of si-STAT1, si-STAT2, and si-STAT3 for 8 h, serum-deprived for 24 h, pretreated with IFN-β (500 U/ml) for 1 h, and stimulated with LPS (1 μg/ml) for 8 h. **(A)** The upper panel shows a representative Western blot image, indicating expression levels of ICAM-1 and GAPDH (used as charge control). The graphical analysis is presented at the bottom of each panel. Error bars indicate the SD for three independent experiments. ^∗∗∗^*p* < 0.001 vs. scramble. ^+++^*p* < 0.001 vs. scramble + LPS. ^###^*p* < 0.001 vs. scramble + IFN-β + LPS. **(B)** The upper panel shows a representative Western blot image, indicating expression levels of VCAM-1 and GAPDH (used as charge control). The graphical analysis is presented at the bottom of each panel. Error bars indicate the SD for three independent experiments. ^∗∗∗^*p* < 0.001 vs. scramble. ^+++^*p* < 0.001 vs. scramble + LPS. ^##^*p* < 0.01 vs. scramble + IFN-β + LPS.

The aim of experiment was to evaluate whether IFN-β-activated STAT proteins could also regulate cell adhesion protein expression in CF (another important characteristic of inflammation). As shown by Figures 5A,B, LPS significantly increased ICAM-1 and VCAM-1 protein expression levels in CF, while prestimulation of IFN-β inhibited LPS-induced expression of ICAM-1 and VCAM-1, by activation of STAT3. On the contrary, neither STAT1 and STAT2 were involved in IFN-β inhibition of cell adhesion proteins expression in LPS-treated CF.

### IFN-β Activates STAT3 to Inhibit LPS-Induced Neutrophil Recruitment by CF

**FIGURE 6:**
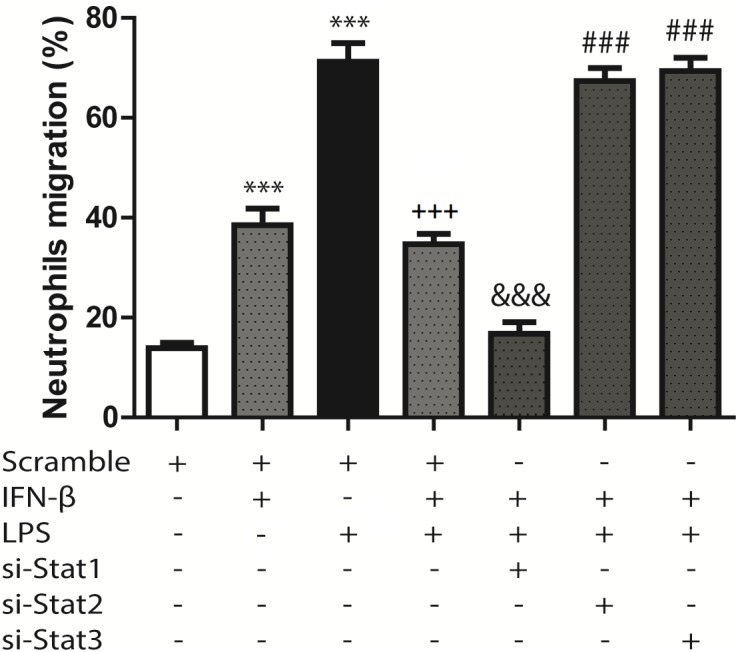
Neutrophil migration induced by conditioned culture media derived from IFN-β- and LPS-treated CF: assays were performed in transwell chambers. Neutrophils charged with fluorescent calcein were seeded in the upper chamber and allowed to migrate at 37°C for 3 h to the lower chamber containing conditioned culture medium derived from CF that were transfected with scramble or 200 ng of STAT1 siRNA, STAT2, and STAT3 for 8 h, serum-deprived for 24 h, pretreated with IFN-β (500 U/ml) for 1 h, and stimulated with LPS (1 μg/ml) for 8 h. Fluorescent neutrophils that migrated to the lower compartment were measured using a fluorescence spectrometer, and the chemotactic activity was expressed as the percentage of fluorescent cells in the lower chamber as compared to the fluorescence of the total neutrophils added. Error bars indicate the SD for three independent experiments. ^∗∗∗^*p* < 0.001 vs. scramble. ^+++^*p* < 0.001 vs. scramble + LPS. ^&&&^*p* < 0.001 vs. scramble + IFN-β. ^###^*p* < 0.001 vs. scramble + IFN-β + LPS.

Previous work from our laboratory has shown that CF are able to recruit immune system cells by secreting chemokines and increasing cell adhesion proteins on the surface of CF. In agreement with our previous findings, we observed that LPS induced IP-10 and MCP-1 secretion in CF, as well as ICAM-1 and VCAM-1 expression, and that IFN-β inhibited these effects. We then asked whether the changes in both cytokines/chemokines and cell adhesion molecules induced by IFN-β could modulate neutrophil recruitment to CF. Neutrophil migration toward conditioned medium obtained from CF treated with IFN-β was increased respect to media from untreated CF. The migration of neutrophils was also increased when moving toward culture media obtained from LPS-treated CF; however, when CF were pretreated with IFN-β before LPS stimulation a synergic effect was not observed and neutrophil migration was similar to that observed in IFN-β alone (Figure 6). Only STAT1 activation was involved in neutrophil migration to IFN-β-treated CF media, whereas STAT2 and STAT3 were necessary for the migration of neutrophils to culture media obtained from LPS-treated CF that were previously incubated with IFN-β (Figure 6).

**FIGURE 7:**
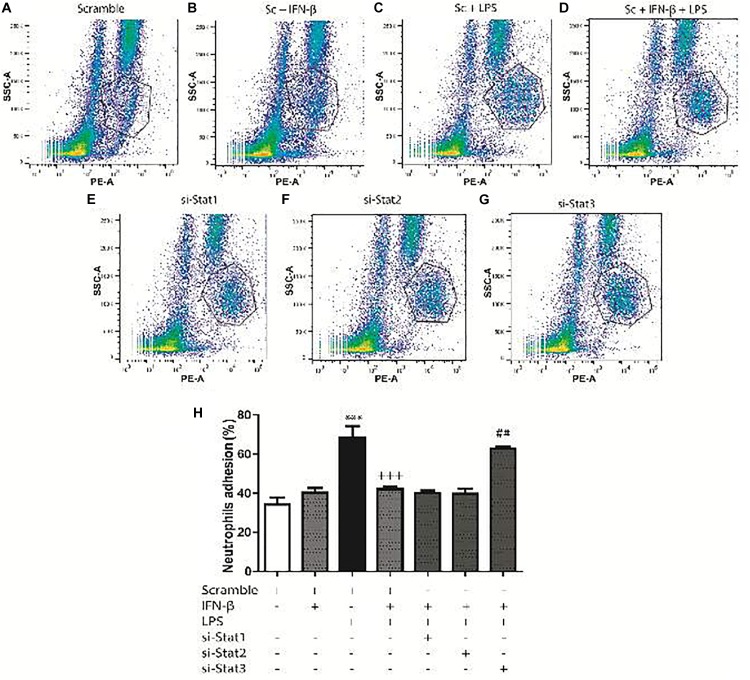
IFN-β decreases neutrophil adhesion to CF. Leukocytes were labeled with anti-RP1/PE (neutrophil marker) and incubated for 2 h at 37°C with confluent monolayers of CF transfected with scramble or 200 ng si-STAT and treated with IFN-β and LPS. Cells were analyzed using flow cytometry. **(A–G)** FACS dots plots are representative images of the percentage of neutrophils adhered to CF. **(H)** Graphical analysis of the percentage of neutrophils in co-culture adhered to scramble-transfected CF with no treatment or treated with IFN-β or LPS. The neutrophil adhesion index was calculated was as follows: 100 x [no. cells (RP1 +) adhered/total number of cells (RP-1 +)]. Error bars indicate the SD for three independent experiments. ^∗∗∗^*p* < 0.001 vs. scramble. ^+++^*p* < 0.001 vs. scramble + LPS. ^##^*p* < 0.01 vs. scramble + IFN-β + LPS.

For the adhesion assays, 34.3% of the neutrophils adhered to CF surface (Figure 7A). Adhesion increased to 40.3% for IFN-β-treated CF (Figure 7B) and to 68.3%for LPS-treated CF (Figure 7C). In a similar trend to our previous results, CF pretreatment with IFN-β before LPS, seems to exert a non-synergic antinflammatory effects since neutrophil adhesion was lower than LPS stimulation alone (42 versus 68.3%, respectively) (Figure 7D). Adhesion was not dependent on STAT1 nor STAT2; however, activation of STAT3 by IFN-β, was crucial for inhibiting the LPS-induced neutrophil adhesion (Figures 7E,F). si-STAT3 blocked the effect of IFN-β, boosting neutrophil adhesion to 62.6% (Figure 7G). Figure 7H shows a graphical analysis of the adhesion results.

## Discussion

IFN-β through differential STAT protein activation, either by itself or previous to a pro-inflammatory stimulus (in this case, LPS), acts as a key regulator of the cellular inflammatory response in CF. The proinflammatory actions of IFN-β are STAT1-dependent, while its anti-inflammatory activity is mediated by STAT2 and/or STAT3 activation.

### IFN-β Activates the JAK/STAT Signaling Pathway in CF

Activation of different STAT proteins by IFN-β produces diverse effects in various cell types (van Boxel-Dezaire et al., 2006). In cardiac tissue some studies have shown that cardiomyocytes express higher levels of IFN-β than CF; however, CF express higher levels of IFNAR, making CF more sensitive than cardiomyocytes to stimulation by IFN-β (Zurney et al., 2007). Type I interferons such as IFN-β can activate all known STAT proteins, and in this study, IFN-β activated STAT1, STAT2, and STAT3 proteins in CF. These actions occurred through JAK, since the effects were inhibited by the JAK inhibitor Ruxolitinib. Zurney et al. (2007), demonstrated that IFN-β induces STAT1 and STAT2 phosphorylation in rat CF (Zurney et al., 2007); however, in CF STAT protein activation is not exclusive to IFN, as IL-6 and Angiotensin II also induce STAT3 activation (Tsai et al., 2008; Meléndez et al., 2010). Our findings show that IFN-β exerts its effects in CF in a time- and concentration-dependent manner. These results are consistent with Pertsovskaya et al. (2013), who demonstrated that IFN-β activates early STAT1 phosphorylation, although additional phosphorylation is observed at later times. Similar results have also been found in other cell types (van Boxel-Dezaire et al., 2006; Happold et al., 2014). Collectively, our results show that IFN-β triggers STAT1, STAT2, and STAT3 protein phosphorylation in CF, with comparable kinetics for all three STAT proteins, at similar IFN-β concentrations and with similar time courses.

### IFN-β Through STAT1 Activation Induce a Pro-inflammatory Effect in CF

To date, there has been little published evidence regarding modulation of cytokine and chemokine secretion by IFN-β in cultured CF. Our results demonstrate a dual action of IFN-β increasing proinflammatory and also anti-inflammatory cytokine secretion; however, previous to a pro-inflammatory environment (such as TLR4 activation by LPS), it behaves as an anti-inflammatory agent.

Chemokines, including MCP-1 and IP-10, are crucial for assembling the immune response, acting as chemoattractants for various immune cells (Frangogiannis, 2004). Our results show that IFN-β increased MCP-1 and IP-10 secretion by activating STAT1. Cumulative evidence suggests that STAT1 activation promotes the release of IP-10, in murine macrophages (Park et al., 2015), and it is well-known that increased STAT1 phosphorylation induces p65-NF-κβ phosphorylation and acetylation, promoting its activation and therefore increasing p65-NF-κβ-dependent proinflammatory response (Furrer et al., 2016). Whereas other studies have showed different results regarding the effect of STAT activation on chemokine and cytokine secretion. In human umbilical vein endothelial cells, MCP-1 mRNA expression was dependent on STAT3 activation (Wiejak et al., 2013). According to our results, we suggest that STAT1 activation by IFN-β plays a key role in the pro-inflammatory response of cultured CF.

### In CF Previous to a Pro-inflammatory Stimulus, IFN-β Through STAT2 and/or STAT3 Activation Induces an Anti-inflammatory Effect

Our results show that IFN-ß by itself, produces a modest increase in secretion of cytokine IL-10. However, when CF are challenged with a potent pro-inflammatory stimulus like LPS, IFN-ß shows a stronger anti-inflammatory effect, as IFN-β inhibited LPS-induced IL-6 and TNF-α secretion. Our results suggest that STAT3 activation is crucial for IFN-β-induced IL-10 secretion, as well as its suppressive effect on the LPS-induced TNF-α and IL-6 secretion. Studies in other cell types partially coincide with our results. Ramgolam et al. (2009), suggested that IFN-β1 induces IL-10 secretion through STAT1 and STAT3 activation, and this effect inhibits Th17 cell differentiation (Ramgolam et al., 2009). However, other studies have found results contrary to ours. In murine dendritic cells, IFN-β-induced IL-10 secretion is mediated through the IFNAR in a STAT2-dependent manner (Yen et al., 2015), whereas STAT1 was also identified as a negative regulator of IL-10 expression in monocytes *in vivo* (VanDeusen et al., 2006). Our results suggest that in CF, IFN-β previous to a pro-inflammatory stimulus, provides a protective, anti-inflammatory effect increasing IL-10 levels and inhibiting LPS-induced IL-6 and TNF-α secretion.

Various studies have shown that CF are the main source of IL-1β in the infarcted myocardium (Long, 2001; Nian et al., 2004). Boza et al. (2016) demonstrated that in CF, LPS increases pro-IL-1β expression, which is dependent on the PI3K/Akt and NF-κβ signaling pathways. However, LPS does not activate the NLRP3 inflammasome and consequently does not triggers pro-IL-1β fragmentation and active IL-1β secretion. The same authors demonstrated that ATP is required for inflammasome assembly and activation, which triggers fragmentation of pro-IL-1β to its active form IL-1β and stimulates its secretion (Boza et al., 2016). Our results show that IFN-β inhibits LPS-induced pro-IL-1β expression through the JAK/STAT2 and/or STAT3 pathways. Although the exact mechanism remains unknown, there is evidence that IFN-β affects IL-1β secretion. Guarda et al. (2011), reported that IFN-I inhibits IL-1β production in bone marrow-derived murine macrophages through transcription of p-STAT1 dimers, repressing the activity of the inflammatory proteins NLRP1 and NLRP3 (Guarda et al., 2011). These interesting findings are consistent with our results, suggesting that IFN-I is capable of inhibiting pro-IL-1β production. The STAT-activated proteins implicated in the studies are different, likely attributable to the different cell types. However, other data from immune cell studies show contradictory results. Malireddi and Kanneganti (2013), demonstrated that IFN-I activates the inflammasome through upregulation of caspase-1 and expression of NLRP3, RIG-I, and AIM2, potentiating the inflammatory response. Given the importance of CF in IL-1β expression and secretion, our results suggest that in CF, the IFN-β through STAT2 and STAT3 activation negatively regulates the inflammatory response triggered by a pro-inflammatory stimulus.

### IFN-β Activates STAT2 and STAT3 to Decrease Cell Adhesion Molecule Expression and Neutrophil Recruitment

Immune cell recruitment is a key step in the progression of inflammation-associated cardiovascular pathologies. This step depends initially on chemokine secretion and ICAM-1 and VCAM-1 expression at the inflammation site (Kukielka et al., 1993; Cook-Mills et al., 2011). Couture et al. (2009), have shown that TNF-α and PMA increase neutrophil recruitment by CF. Previous results from our laboratory have shown that in CF, TLR4 activation by LPS, induces monocyte recruitment by CF themselves, due initially to the monocyte migration favored by MCP-1 secretion; whereas ICAM-1 and VCAM-1 expression in turn favors monocyte adhesion (Humeres et al., 2016). Our results show that in CF, IFN-β activates STAT3 to inhibit LPS-induced ICAM-1 and VCAM-1 expression. However, other studies have reported different results for the effect of IFN-β on adhesion protein expression. Combined treatment with TNF-α, IL-1β, and IFN-β has been shown to increase ICAM-1 and VCAM-1 expression in human umbilical vein endothelial cells (Shen et al., 1997; Dhib-Jalbut and Marks, 2010). Similarly, in the alveolar macrophages of rats with bleomycin-induced pulmonary fibrosis, STAT1 activation correlated significantly with increased ICAM-1 expression and severity of lung tissue inflammation (Fan and Wang, 2005). Collectively, our results reinforce the concept that STAT2 and/or STAT3 activation by IFN-β are essentials for triggering the anti-inflammatory response in CF.

Neutrophil infiltration occurs within the first few hours after tissue injury (Braunwald and Kloner, 1985), representing a crucial step for initiation of inflammatory repair (Kakkar and Lefer, 2004; Frangogiannis, 2006). Our results show that in CF, treatment with IFN-β alone increases neutrophil migration through STAT1 activation. There is abundant and contradictory data in the literature regarding the effects of IFN-β on cell migration. IFN-β prevents neutrophil infiltration in animal models of cerebral inflammation (Veldhuis et al., 2003a,b), and decreases serum-induced migration of polymorphonuclear cells (Aas et al., 1996). On the other hand, our results show that when CF are challenged by a pro-inflammatory stimulus such as LPS, IFN-β through STAT2 and STAT3 activation decreases both chemokine secretion and adhesion molecule expression. This effects decreases neutrophil recruitment, demonstrating the anti-inflammatory properties of IFN-β in CF.

To date, no other *in vitro* studies have elucidated the effect of IFN-β and/or STAT activation on neutrophil recruitment by CF. Our results allow us to conclude that previous to a pro-inflammatory environment induced by an inflammatory insult, IFN-β exerts an anti-inflammatory effect in CF, reducing neutrophil recruitment. It is well-known that neutrophil recruitment after cardiac injury is crucial for rapidly inducing the healing process; however, prolonged neutrophil recruitment may lead to chronic inflammation in the tissue and eventually cardiac fibrosis (Frangogiannis et al., 2002; Frangogiannis and Entman, 2005; Frangogiannis, 2006).

## Conclusion

**FIGURE 8:**
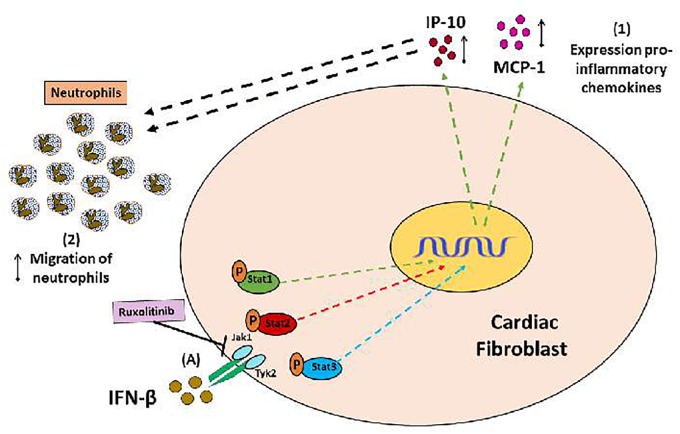
Proinflammatory and anti-inflammatory effects of IFN-β in CF: (A) IFN-β triggers JAK/STATsignaling pathway activation (STAT1, STAT3, and STAT3 proteins). (1) IFN-β induces a pro-inflammatory response in the CF through STAT1 protein activation (green arrows), characterized by increased expression of the chemokines IP-10 and MCP-1, as well as (2) increased neutrophil migration, favored by chemokines released from CF.

**FIGURE 9:**
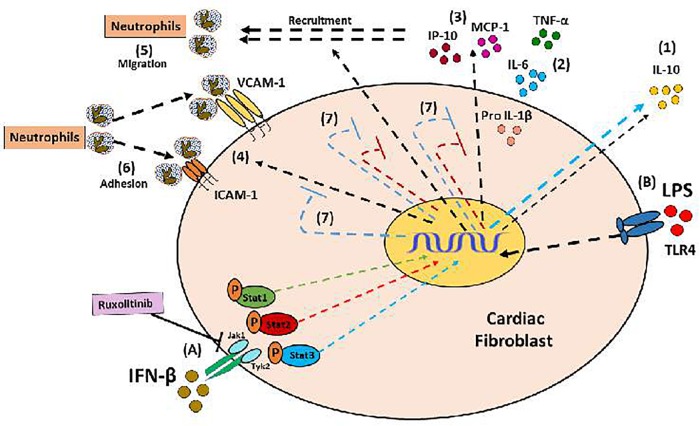
Anti-inflammatory effect of IFN-β in CF: **(A)** IFN-β induces the activation of its JAK/STAT transduction pathway (proteins Stat1, Stat3, Stat3); and directly through Stat3 induces the secretion of IL-10 (1). **(B)** The LPS is a pro-inflammatory stimulus that favors the secretion of cytokines (2), chemokines (3), expression of adhesion molecules (4), which finally favors the recruitment of neutrophils by the FC (5 and 6) . (7) IFN-β through the activation of Stat2 and Stat3 proteins (red and blue STOP lines), inhibits the pro-inflammatory effects of LPS.

Collectively, our results help to understand the *in vitro* regulation of INF-β-mediated inflammatory response in CF. IFN-β plays a dual role in regulating inflammation, acting as a crucial player in modulating the pro- or anti-inflammatory behavior of CF depending of environment. IFN-β by itself, activates STAT1 to generate a pro-inflammatory response, characterized by increased chemokines MCP-1 and IP-10 expression, which increase neutrophil migration (see Figure 8). However, IFN-β also activates STAT3 to induce secretion of the anti-inflammatory cytokine IL-10 (see Figure 9, blue arrow). Nevertheless, previous to a pro-inflammatory environment TLR4-activated, IFN-β triggers a marked anti-inflammatory effect in CF by activating STAT2 and/or STAT3. This anti-inflammatory effect is characterized by inhibition of LPS-induced IL-6, TNF-α, MCP-1, and IP-10 secretion and reduction of pro-IL-1β, ICAM-1, and VCAM-1 expression, decreasing neutrophil recruitment.

## Author Contributions

SB, RA, CH, RV, PB, and GD-A contributed to the conception and design of the study. CM and VP-J organized the database. SB, FO-S, and RV performed the statistical analysis. SB and GD-A wrote the first draft of the manuscript. CM, VP-J, PB, and CH wrote sections of the manuscript. All authors contributed to manuscript revision, read and approved the submitted version.

## Conflict of Interest Statement

The authors declare that the research was conducted in the absence of any commercial or financial relationships that could be construed as a potential conflict of interest.

## Footnotes

1 https://grants.nih.gov/grants/olaw/guide-for-the-care-and-use-of-laboratory-animals.pdf

## Supplementary Material

FIGURE S1 Identification of neutrophil population isolated from bone marrow.

FIGURE S2 Effectiveness and selectivity of siRNA.
