# Supplementary figures and images for: IFN-β Plays Both Pro- and Anti-inflammatory Roles in the Rat Cardiac Fibroblast Through Differential STAT Protein Activation
Source: Front Pharmacol. 2018 Nov 28;9:1368. doi: 10.3389/fphar.2018.01368 (PMC6280699; doi:10.3389/fphar.2018.01368)

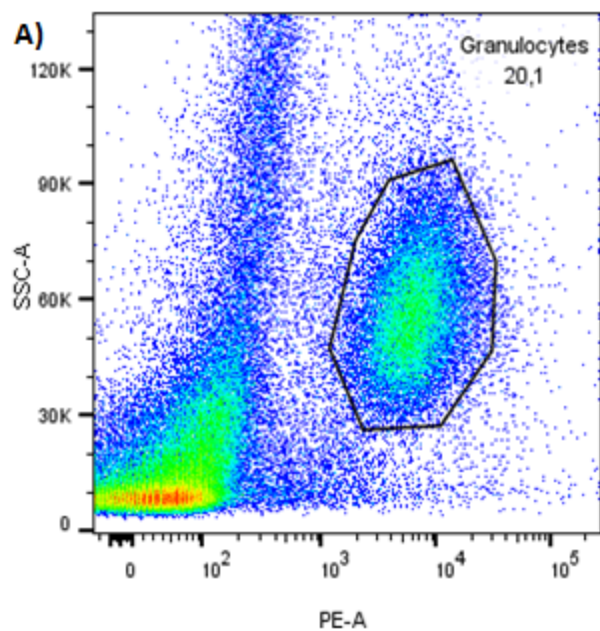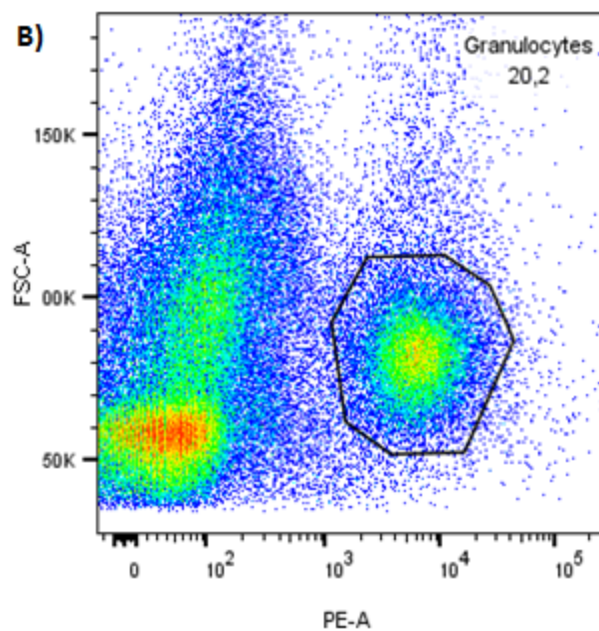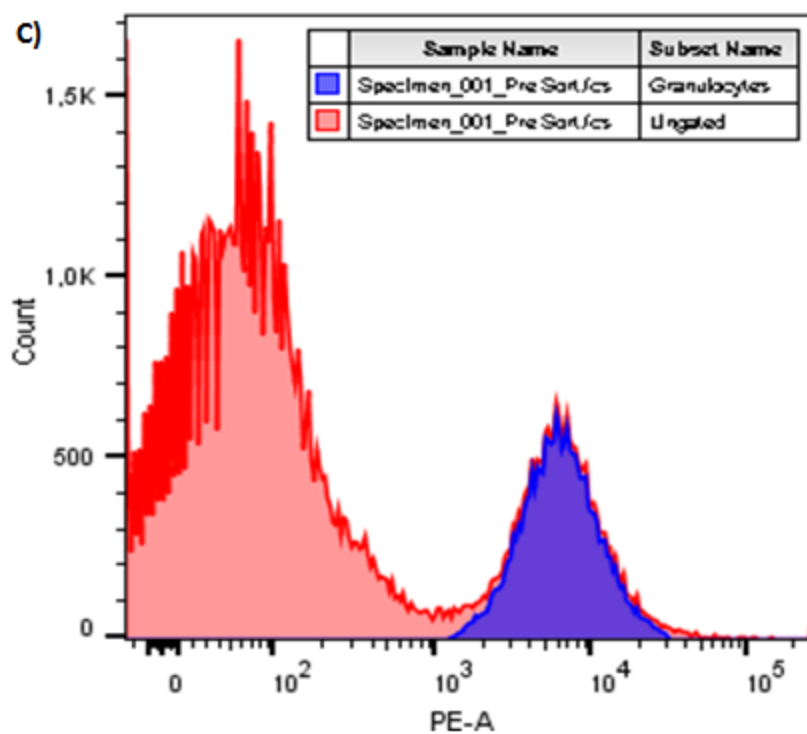

Supplement: FIGURE S1 — Identification of neutrophil population isolated from bone marrow. [file Data_Sheet_1.PDF]

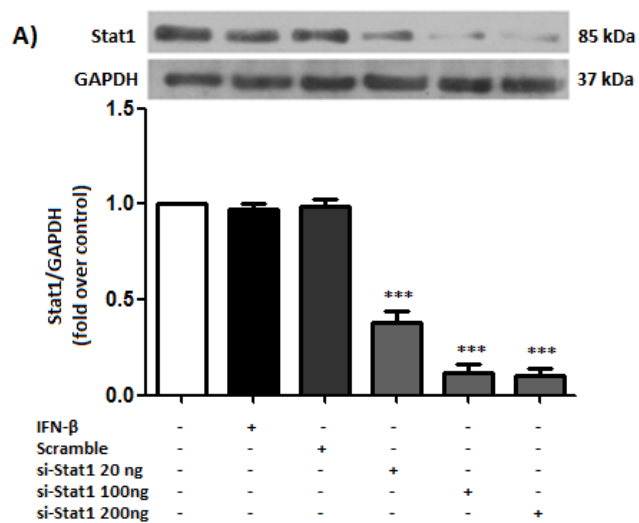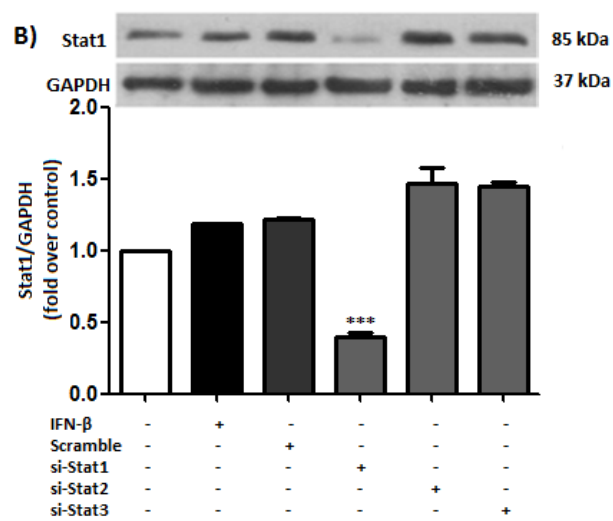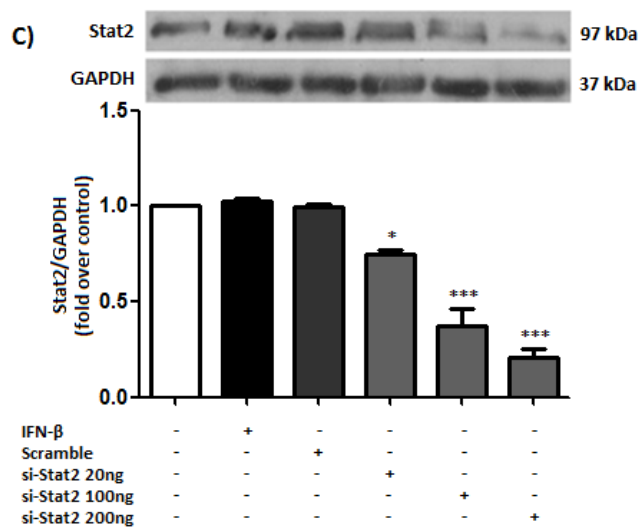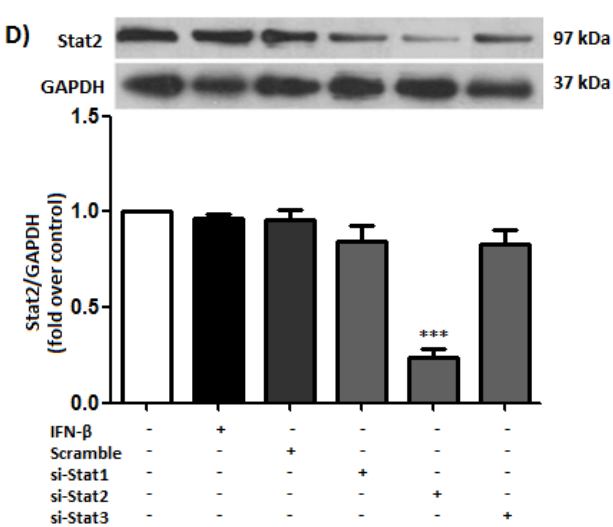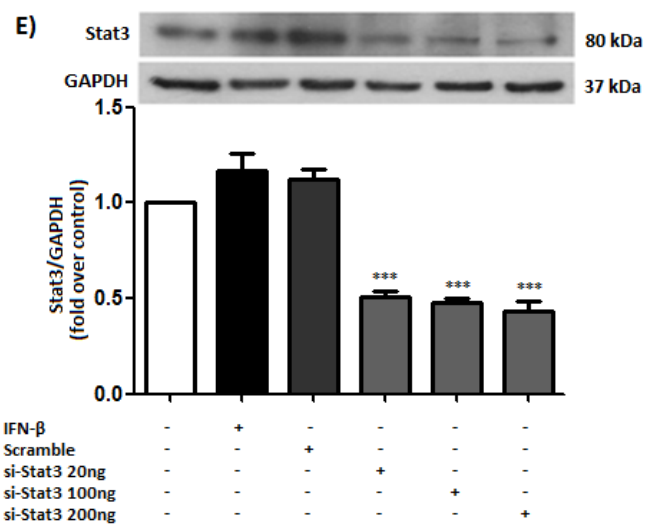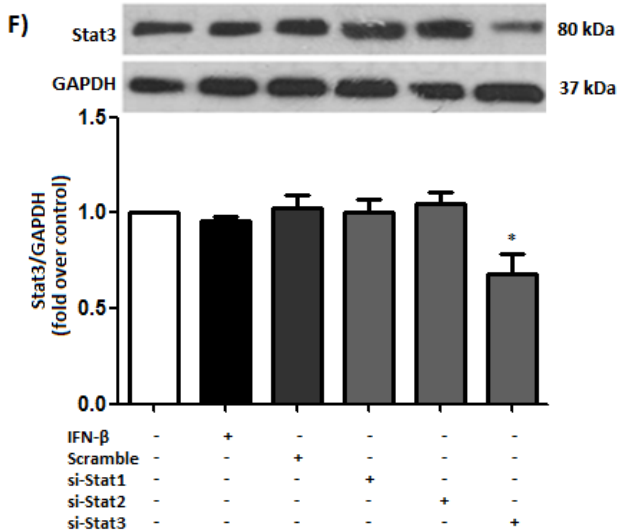

Supplement: FIGURE S2 — Effectiveness and selectivity of siRNA. [file Data_Sheet_2.PDF]
